# Performance of 24-hour urinary creatinine excretion-estimating equations in relation to measured 24-hour urinary creatinine excretion in hospitalized hypertensive patients

**DOI:** 10.1038/s41598-019-40416-w

**Published:** 2019-03-05

**Authors:** Piotr Jędrusik, Bartosz Symonides, Zbigniew Gaciong

**Affiliations:** Department of Internal Medicine, Hypertension and Vascular Diseases, Medical University of Warsaw, Warsaw, Poland

## Abstract

Estimated 24-hour urinary creatinine excretion (24 hrUCr) may be useful for converting spot urine analyte/creatinine ratio into estimated 24-hour urinary excretion of the evaluated analyte, and for verifying completeness of 24-hour urinary collections. We compared various published 24 hrUCr-estimating equations against measured 24 hrUCr in hospitalized hypertensive patients. 24 hrUCr was measured in 293 patients and estimated using eight formulas (CKD-EPI, Cockcroft-Gault, Walser, Goldwasser, Rule, Gerber-Mann, Kawasaki, Tanaka). We used the Pearson correlation coefficient, the Bland-Altman method, and the percentage of estimated 24 hrUCr within 15%, 30% (P30), and 50% of measured 24hUCr to compare estimated and measured 24 hrUCr. Differences between the mean bias by eight formulas were evaluated using the Friedman rank sum test. Overall, the best formulas were CKD-EPI (mean bias 0.002 g/d, P30 86%) and Rule (mean bias 0.022 g/d, P30 89%), although both tended to underestimate 24 hrUCr with higher excretion values. The Gerber-Mann formula and the Asian formulas (Tanaka, Kawasaki) were less precise in our study population but superior in an analysis restricted to subjects with highest measured 24 hrUCr per body weight. We found significant differences between 24 hrUCr-estimating equations in hypertensive patients. In addition, formula performance was critically affected by inclusion criteria based on measured 24 hrUCr per body weight.

## Introduction

Due to inconvenience and possible errors of 24-hour urine collection^1–3^, spot urine analyte/creatinine ratios have been used as a surrogate measure of 24-hour urinary excretion of such analytes as albumin, iodine, or catecholamines^4–6^. Formulas derived from spot urine analyte/creatinine ratios have also been developed that allow estimating 24-hour urinary excretion of sodium and potassium based on spot urine measurements, yielding fair precision of these estimates at least at the population level^7–10^. We have previously shown^11,12^ a potential utility of estimating 24-hour urinary excretion of sodium and potassium using such a formula in patients with hypertension by simply multiplying the spot urine analyte/creatinine ratio by 24-hour urinary creatinine excretion (24 hrUCr).

However, since the ultimate purpose of spot urine testing is to render 24-hour urine collection unnecessary, reasonable estimation of 24 hrUCr is also needed, and the latter varies by age, gender, body weight, ethnicity, and other factors^13–18^. Using the estimated 24 hrUCr, the spot urine analyte/creatinine ratio can be converted into a more comprehensible parameter, i.e., the estimated 24-hour urinary excretion of the evaluated analyte^19^. It has also been suggested that estimated 24 hrUCr may be useful as a measure of the completeness of a given 24-hour urine collection, indicating whether the actual measured 24 hrUCr is consistent with the estimates based on patient demographic and anthropometric characteristics^20^.

A number of equations have been developed to estimate 24 hrUCr based on simple demographic and anthropometric parameters, including age, gender, race, and most commonly body weight. These include formulas developed and reported by Cockcroft and Gault^13^, Walser^14^, Goldwasser *et al*.^16^, Rule *et al*.^21^, and most recently by Ix *et al.*^20^ using data from the Chronic Kidney Disease Epidemiology Collaboration (CKD-EPI) research group and by Gerber and Mann^19^. In addition, 24 hrUCr-estimating equations were also reported for Kawasaki and Tanaka formulas for spot urine-based estimation of 24-hour urinary excretion of potassium and sodium, originally derived in Asian populations^22^. However, the relative performance of these formulas has not been well studied, as only some of them were included in a previously published comparison against measured 24 hrUCr^20^, and no previous study compared all eight formulas. In particular, the performance of these formulas has never been compared in patients with hypertension, in whom the potential utility of estimating 24 hrUCr may include both estimating 24-hour urinary excretion of various analytes, such as sodium and potassium^12^, and assessment of completeness of 24-hour urine collections^20^.

Thus, in the present study we aimed to compare various 24 hrUCr-estimating equations against measured 24 hrUCr, and indirectly against each other, in an unselected patient population admitted to a specialist hypertensive unit and undergoing routine inpatient clinical evaluation which provided an opportunity to obtain 24-hour urine collections for the reference measurement of 24 hrUCr.

## Results

The study population (n = 293) consisted of 170 women and 123 men at the mean age of 54 ± 16 years. All patients were Caucasians. Table 1 shows detailed characteristics of the overall study population. The diagnosis of sustained hypertension was made in 93% of patients, and 91% of patients were on chronic antihypertensive drug therapy. Most of those without sustained hypertension were found to have white coat hypertension only and some had only episodic hypertension (i.e., episodic blood pressure elevations but no sustained hypertension). For the same reasons, some patients did not receive antihypertensive medications, but all patients received advice regarding non-drug treatment.

**Table 1:** Characteristics of the overall study population (all patients).

Variable	Study population (n = 293)
Gender (male/female)	123/170
Age, years – mean ± SD (range)	54 ± 16 (16–94)
Race/ethnicity – Caucasians	100%
Body mass index, kg/m^2^ – mean ± SD	28.9 ± 5.4
Sustained hypertension*, n (%)	272 (93%)
Cardiovascular disease, n (%)	87 (30%)
Diabetes mellitus, n (%)	55 (19%)
Clinic BP, mm Hg – mean ± SD	153 ± 29/92 ± 16
Ambulatory daytime BP, mm Hg – mean ± SD	132 ± 18/78 ± 12
Ambulatory night-time BP, mm Hg – mean ± SD	121 ± 21/69 ± 12
Any hypertensive medication, n (%)	267 (91%)
24-hour urinary creatinine excretion, g/d – mean ± SD	1.25 ± 0.5
24-hour urinary creatinine excretion, mg/kg/d – mean ± SD	15.3 ± 5.0
Serum creatinine, mg/dL – mean ± SD	0.94 ± 0.46
Estimated GFR <60 mL/min/1.73 m^2^, n (%)	44 (15%)

BP, blood pressure; GFR, glomerular filtration rate (estimated using the Modification of Diet in Renal Disease [MDRD] equation); SD, standard deviation.

Data are presented as n (%), mean ± SD, or mean ± SD (range) as indicated.

*Excluding subjects with white coat hypertension or only episodic blood pressure elevation.

**Figure 1:**
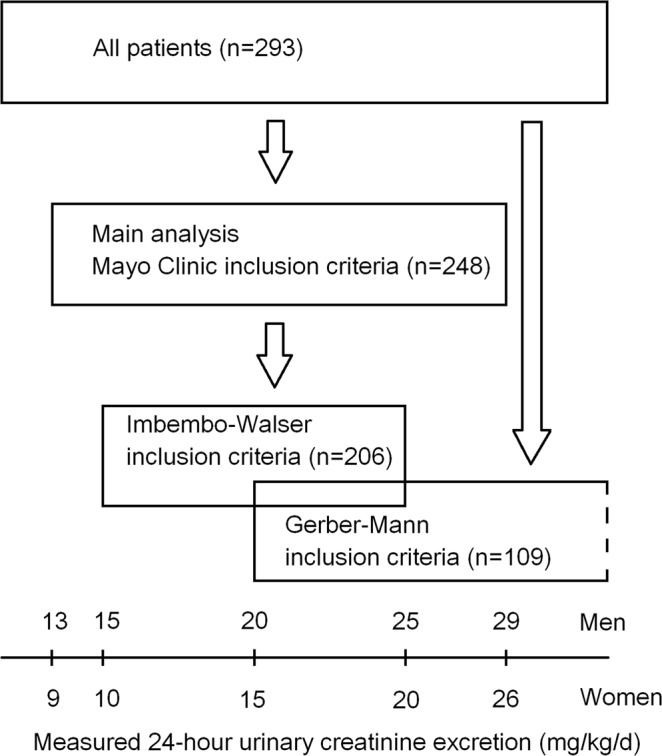
Flowchart showing the number of patients in each analysis. The main analysis was performed in patients who met the inclusion criteria for measured 24-hour urinary creatinine excretion (24 hrUCr) by the Mayo Clinic^28^ (13–29 mg/kg/d in men, 9–26 mg/kg/d in women; n = 248). Additional (sensitivity) analyses were performed in all patients (overall study population, n = 293) and in subsets defined by alternative inclusion criteria by Imbembo and Walser^29^ (24 hrUCr 15–25 mg/kg/d in men, 10–20 mg/kg/d in women; n = 206) and by Gerber and Mann^19^ (24 hrUCr ≥ 20 mg/kg/d in men, ≥15 mg/kg/d in women; n = 109).

In the overall study population, the mean 24 hrUCr was 1.25 ± 0.5 g/d (range 0.29–2.83 g/d), or 15.3 ± 5.0 mg/kg/d (range 3–32 mg/kg/d). The inclusion criteria for measured 24 hrUCr by the Mayo Clinic were met by 248 patients who were included in the main analysis. The alternative inclusion criteria by Imbembo and Walser were met by 206 patients, and the alternative inclusion criteria by Gerber and Mann were met by 109 patients (Fig. 1).

Estimated 24 hrUCr correlated strongly with measured 24 hrUCr, especially with more restrictive inclusion criteria for measured 24 hrUCr (R = 0.66 to 0.73 in the overall study population [n = 293], R = 0.72 to 0.84 with the Mayo Clinic inclusion criteria [main analysis, n = 248], R = 0.79 to 0.89 with the alternative inclusion criteria by Imbembo and Walser [n = 206], and R = 0.84 to 0.93 with the alternative inclusion criteria by Gerber and Mann [n = 109]) (all P < 0.001) (Table 2 and Supplementary Table).

**Table 2:** Performance of 24-hour urinary creatinine excretion (24 hrUCr)-estimating formulas.

Formula	Bias (g/d)	95% CI	AbsDiff	R	95% LoA	P15	P30	P50
**Main analysis – Mayo Clinic inclusion criteria** ^28^ **(n = 248)**								
CKD-EPI	0.002	−0.031 to 0.039	0.209	0.83	−0.523 to 0.527	54	86	98
Cockcroft-Gault	0.041	−0.002 to 0.077	0.231	0.79	−0.546 to 0.628	52	81	98
Walser	−0.071	−0.114 to −0.036	0.227	0.81	−0.634 to 0.492	56	82	94
Goldwasser	−0.055	−0.102 to −0.012	0.266	0.72	−0.712 to 0.603	42	75	91
Rule	0.022	−0.013 to 0.057	0.210	0.83	−0.511 to 0.555	56	89	98
Gerber-Mann	−0.305	−0.344 to −0.267	0.347	0.81	−0.868 to 0.259	33	56	79
Tanaka	−0.221	−0.262 to −0.181	0.316	0.76	−0.837 to 0.394	40	63	84
Kawasaki	−0.124	−0.164 to −0.096	0.239	0.84	−0.677 to 0.428	53	80	94
Alternative inclusion criteria* by Gerber and Mann ^19^ **(n = 109)**								
CKD-EPI	0.233	0.200 to 0.279	0.240	0.93	−0.162 to 0.628	53	94	100
Cockcroft-Gault	0.236	0.182 to 0.282	0.277	0.87	−0.254 to 0.725	49	82	100
Walser	0.144	0.100 to 0.188	0.203	0.90	−0.284 to 0.573	62	97	100
Goldwasser	0.182	0.124 to 0.238	0.261	0.84	−0.373 to 0.737	53	92	100
Rule	0.244	0.200 to 0.287	0.257	0.92	−0.207 to 0.696	55	95	100
Gerber-Mann	−0.043	−0.078 to −0.002	0.155	0.93	−0.404 to 0.318	74	99	100
Tanaka	0.028	−0.021 to 0.078	0.197	0.87	−0.474 to 0.529	68	95	100
Kawasaki	0.073	0.034 to 0.113	0.169	0.92	−0.321 to 0.467	78	99	100

CKD-EPI – Chronic Kidney Disease Epidemiology Collaboration; Bias – measured minus estimated 24 hrUCr; 95% CI – 95% confidence interval (Friedman rank sum test); AbsDiff – average of absolute differences between measured and estimated 24 hrUCr (g/d); R – Pearson correlation coefficient (estimated vs. measured 24 hrUCr); 95% LoA – 95% limits of agreement (Bland-Altman method) (g/d); P15, P30, P50 – percentage of estimated 24 hrUCr values within 15%, 30%, 50% of actual measured 24 hrUCr.

*Results of other sensitivity analyses (in all patients and the patient subset defined by the alternative inclusion criteria by Imbembo and Walser) – see Supplementary Table.

**Figure 2:**
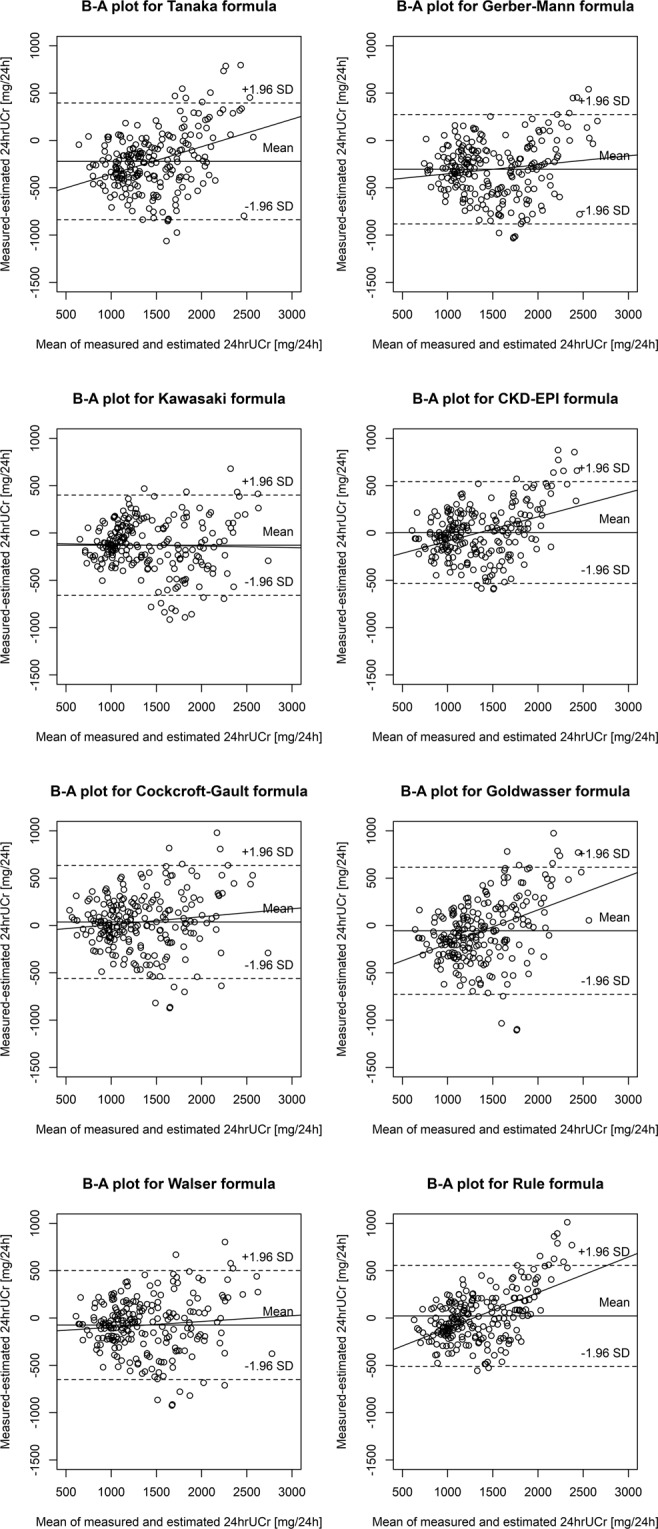
Bland-Altman (B-A) plots showing the difference between measured and estimated 24-hour urinary creatinine excretion (24 hrUCr) plotted against the mean 24 hrUCr by the two methods in the main analysis (inclusion criteria by the Mayo Clinic; n = 248). CKD-EPI, Chronic Kidney Disease Epidemiology Collaboration; SD, standard deviation.

In the main analysis (inclusion criteria by the Mayo Clinic, n = 248; Table 2 and Fig. 2), the mean bias was lowest for the CKD-EPI formula (0.002 g/d), with 95% of the individual estimations within ± 0.53 g/d compared to the measured value, and P30 of 86%. However, based on visual inspection of the regression line on the Bland-Altman plot, the CKD-EPI formula tended to underestimate 24 hrUCr with higher excretion values. Very similar results were obtained for the Rule formula (mean bias 0.022 g/d, 95% limits of agreement −0.51 to +0.55 g/d, P30 89%). This formula also tended to underestimate 24 hrUCr with higher excretion values. Other older formulas (Cockcroft-Gault, Walser, Goldwasser) were slightly worse in terms of the mean bias, 95% limits of agreement, and P30. However, except for the Goldwasser formula, these formulas did not underestimate 24 hrUCr with higher excretion values. Formulas developed in Asian populations (Tanaka, Kawasaki) were inferior in our study population, especially the Tanaka formula (mean bias −0.22 g/d, P30 63%). The Gerber-Mann formula showed the highest mean bias (−0.30 g/d) and lowest P30 (56%). The comparison based on the average of absolute differences between measured and estimated 24 hrUCr for each formula yielded similar results to that based on the mean bias and P30, as the lowest values were obtained for the CKD-EPI and Rule formulas, and the highest value for the Gerber-Mann formula. Overall differences between formulas were significant by the Friedman rank sum test (P < 0.00001).

In our sensitivity analyses, we obtained similar results in all patients (overall study population) and with the alternative inclusion criteria by Imbembo and Walser, as in both these analyses the relative performance of the compared formulas was generally consistent with the main analysis, with the CKD-EPI and Rule formulas being the most precise (in terms of the mean bias, the average of absolute differences between measured and estimated 24 hrUCr, 95% limits of agreement, and P30), and the Gerber-Mann formula being the least precise (Supplementary Table). However, all formulas tended to be less precise in the overall study population (all patients), as evidenced by consistently higher bias and the average of absolute differences between measured and estimated 24 hrUCr, wider 95% limits of agreement, and smaller P30 values compared to the main analysis, and more precise in the subset defined by the alternative inclusion criteria by Imbembo and Walser, as evidenced by slightly smaller or similar bias values, smaller average of absolute differences between measured and estimated 24 hrUCr, narrower 95% limits of agreement, and higher P30 values compared to the main analysis (Supplementary Table).

**Figure 3:**
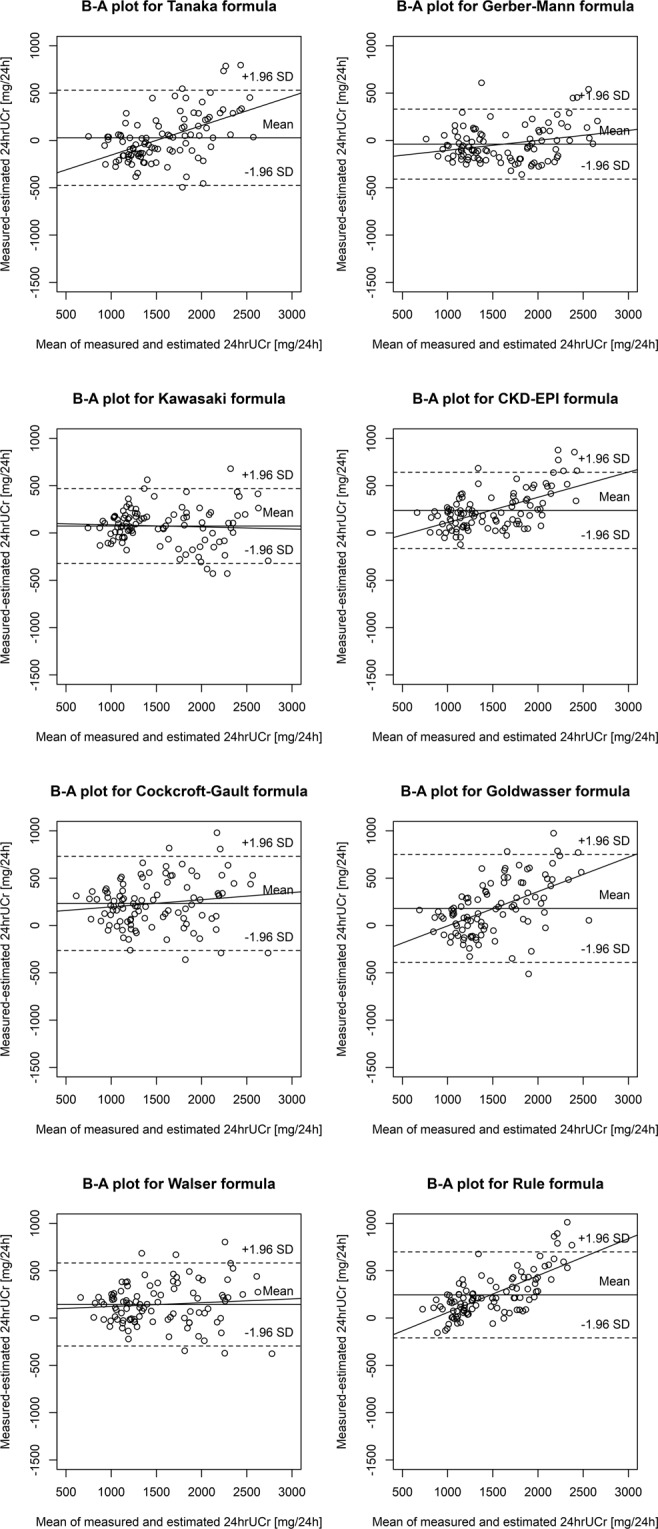
Bland-Altman (B-A) plots showing the difference between measured and estimated 24-hour urinary creatinine excretion (24 hrUCr) plotted against the mean 24 hrUCr by the two methods in the patient subset defined by the alternative inclusion criteria by Gerber and Mann (n = 109). CKD-EPI, Chronic Kidney Disease Epidemiology Collaboration; SD, standard deviation.

In contrast, different results (Table 2 and Fig. 3) were obtained in the patient subset selected using the alternative inclusion criteria by Gerber and Mann, in which the Gerber-Mann formula was the best (mean bias −0.04 g/d, P30 99%). The only other formula that was similarly accurate in this subset was the Kawasaki formula, while the Tanaka formula was characterized by an even smaller mean bias (0.03 g/d) but with wider 95% limits of agreement and a higher average of absolute differences between measured and estimated 24 hrUCr. For all other formulas, the results were worse, with significant underestimation of 24 hrUCr by the CKD-EPI and Rule formulas (mean bias at least 0.23 g/d), although P30 values were high for all formulas. Similarly to the main analysis, the comparison based on the average of absolute differences between measured and estimated 24 hrUCr for each formula yielded results consistent with that based on the mean bias, and overall differences between formulas were significant by the Friedman rank sum test (P < 0.00001).

## Discussion

In the present study, we focused on the comparative performance of various 24 hrUCr-estimating formulas in hospitalized hypertensive patients, confirming their utility in this population, but also showing significant differences between the formulas.

For comparison of estimated versus measured 24 hrUCr for each formula, we calculated several parameters, including the Pearson correlation coefficient, the mean bias and 95% limits of agreement by the Bland-Altman method^23,24^, the average of absolute differences between measured and estimated 24 hrUCr, and the percentage of estimated 24 hrUCr within 15% (P15), 30% (P30), and 50% (P50) of the measured 24 hrUCr. Of the latter three parameters, we considered P30 as the most clinically useful threshold for the errors of individual 24 hrUCr estimation using various formulas. In previous studies, P30 was a commonly used criterion to evaluate accuracy of glomerular filtration rate (GFR)-estimating equations^25,26^, thereby providing a reference for comparisons between various estimating equations.

Evaluation of the precision and bias of 24 hrUCr-estimating formulas depends on whether the reference 24-hour urine sample was collected accurately. Both under- and overcollection of 24-hour urine may occur^1–3^, and undercollection was reported to affect as many as 30% of collections^27^. To reduce bias related to potential 24-hour urine under- or overcollection, we included only patients in whom the measured 24 hrUCr was within the expected ranges, indicating completeness of the 24-hour urine collection. However, various reference ranges of the actual measured 24 hrUCr were reported in the literature^28,29^, and studies in which the evaluated formulas were developed also used different inclusion/exclusion criteria based on the actual measured 24 hrUCr^19,20^. Thus, in addition to the main analysis using the inclusion criteria by the Mayo Clinic^28^, we performed a sensitivity analysis, repeating our formula comparison in all patients and in two subsets defined using alternative inclusion criteria by Imbembo and Walser^29^ and by Gerber and Mann^19^.

In our main analysis, the best formulas were the CKD-EPI formula and the Rule formula, while the Gerber-Mann formula was clearly inferior, as were the Asian formulas (Tanaka and Kawasaki). The main drawback of the CKD-EPI and Rule formulas was that both tended to underestimate 24 hrUCr with higher excretion values, as inferred from visual inspection of the slope of the Bland-Altman plot regression lines. Two of the older formulas (Cockcroft-Gault and Walser) were slightly worse in terms of the mean bias, the average of absolute differences between measured and estimated 24 hrUCr, 95% limits of agreement, and P30 but did not underestimate 24 hrUCr with higher excretion values. The results of formula comparison in all patients and in the subset defined by the alternative inclusion criteria by Imbembo and Walser^29^ were generally similar to those of the main analysis. Different results were obtained, however, when we compared various formulas in patients selected using the alternative inclusion criteria by Gerber and Mann^19^, restricting the study population to those subjects with highest 24 hrUCr in relation to body weight. In this relatively small subset of patients, the best formula was the Gerber-Mann formula, followed by the Asian formulas, while other formulas were much less precise.

Our results seem to indicate that at the individual level, precision of estimating 24 hrUCr in hypertensive patients using the best formulas, although not ideal due to underestimation with higher excretion values, is in general similar to the precision of established clinical tools such as GFR-estimating equations^20,25^ which are considered sufficiently precise for clinical use. In addition, the observed agreement between estimated and measured 24 hrUCr indicates that in hypertensive patients, estimation of 24 hrUCr using formulas based on demographic and anthropometric variables may indeed facilitate assessment of completeness of 24-hour urine collections (performed for whatever clinical reasons)^20^.

The observed differences in performance between various formulas may be related to methodological differences between these formulas, including the choice of demographic and anthropometric variables, size and ethnic composition of a sample used to derive a formula, renal function in the development cohort, existence of an independent validation cohort, and reliability of the reference 24-hour urine collection^13,14,16,19–21^. Of the two best formulas in our study, one (Rule) was developed in 664 healthy non-Hispanic white subjects from the United States (US) general population (mean age 55 ± 20 years, 51% women) with generally normal renal function (mean serum creatinine level 0.81 ± 0.24 mg/dl, 8.9% of subjects with GFR <60 mL/min/1.73 m^2^)^21^. No data on the prevalence of hypertension were given but it can be expected to be similar to that reported in the general US population. The other best formula (CKD-EPI) was developed using data collected in 1644 patients from three US kidney disease trials (mean age 44 ± 14 years, 41% women, 11% black, median serum creatinine level 1.4 mg/dl) but with a wide range of GFR values (including 40% of subjects with GFR >60 mL/min/1.73 m^2^, 60% with GFR <60 mL/min/1.73 m^2^, and 25% with GFR <30 mL/min/1.73 m^2^)^20^. No detailed data on the rate of hypertension in these subjects were given, but the original trials were performed in populations with diabetes, chronic kidney disease (CKD) and/or diabetic nephropathy, so the proportion of hypertensives may be expected to be higher compared to the general population but less than in our study population. Thus, compared to the populations in which the CKD-EPI and Rule formulas were developed, our study population was generally similar in regard to age, female/male ratio, and racial characteristics, while the proportion of patients with kidney dysfunction in our study population was lower compared to the population studied by Ix *et al*. (CKD-EPI equation)^20^ but higher compared to the general population sample in the study by Rule *et al*.^21^.

Tanaka and Kawasaki equations were developed in healthy Asian populations^7,8^ but their corresponding equations for potassium and sodium have been a subject of much research also in non-Asian populations^22,30^. Notably, the Kawasaki formula was chosen for spot urine measurement-based estimation of 24-hour urinary excretion of sodium and potassium in a number of large worldwide observational analyses to evaluate the relation between urinary sodium and potassium and cardiovascular events^31–33^ but in our study population, the precision of its 24 hrUCr-estimating component was inferior compared to a number of other formulas.

Of particular interest is the Gerber-Mann formula, as it was developed in a population that was very similar to our patients in regard to age, body mass index, male/female ratio, renal function, and a high proportion of hypertensives, it produced results different from many other formulas, and its development was based of specific methodological assumptions that likely affected our results with this formula. Gerber and Mann^19^ used restrictive inclusion criteria for the actual measured 24 hrUCr, presumed to indicate completeness of 24-hour urine collections. These criteria, however, refer to healthy young men and women^34^ and not necessarily apply equally well to a middle aged, overweight and mostly hypertensive population, as in our study and the study by Gerber and Mann^19^. It is thus possible that Gerber and Mann selected not only complete collectors, as intended, but also those with other characteristics, such as younger age, better preserved renal function, or higher muscle mass^14,21^ in comparison to populations in which other formulas were developed. Our findings indicate that if a formula was developed using such restrictive subject inclusion criteria, based on data collected in subjects with highest 24 hrUCr per body weight (regardless of whether the latter truly reflected completeness of 24-hour urine collection, or was related to other subject characteristics), it may not necessarily perform equally well in those with on average lower 24 hrUCr per body weight, such as the hypertensive population in our study.

However, as indicated by Gerber and Mann, the problem of incomplete collections while developing the equations was perhaps indeed not adequately addressed in the previous studies^19^. Of the two best formulas in our study, the CKD-EPI formula was developed using very liberal inclusion criteria for reference 24 hrUCr^20^, and the Rule formula was developed using outpatient 24-hour urine collections as a reference, and the authors provided no information on the assessment of completeness of these urine collections^21^. On the other hand, it can be argued that 24-hour urine collection completeness criteria by Gerber and Mann might have been actually overly restrictive. Due to inpatient settings of our study, it seems unlikely that only 37% of patients would provide complete 24-hour urine collection, as should be inferred based on these criteria.

Our results can be compared with previously published comparisons of some of these formulas, mostly performed in CKD patients^20,35^ but none of them including the Gerber-Mann formula and the Asian formulas. Five formulas were included in the study by Ix *et al*.^20^ who developed the CKD-EPI formula. In their study, the mean bias for the CKD-EPI formula was −10 mg/d, and P30 was 79%, which is generally similar to our estimates. For the other four formulas evaluated in that study (Cockcroft-Gault, Walser, Goldwasser, and Rule) the mean bias ranged from −28 mg/d to 63 mg/d, and P30 values ranged from 76% to 81%, again very similar results to our findings.

To the best of our knowledge, various 24 hrUCr-estimating formulas have never been compared in patients with hypertension. However, these formulas are potentially useful in hypertensive patients for two major reasons, which justifies studying them in a hypertensive cohort. First, their use might reduce or eliminate the need for performing 24-hour urine collection to measure excretion of some parameters that are commonly evaluated in hypertensive patients (such as 24-hour urinary sodium or potassium excretion), as spot urine measurements might be then used for that purpose instead of a 24-hour urine collection^12^. Second, if a 24-hour urine collection is performed in such patients for any clinical reason, there is a need to verify whether the collection has been complete, and this is not always easily achieved in clinical practice (e.g., when the patient collects 24-hour urine on an outpatient basis). In this regard, estimation of 24 hrUCr using formulas based on demographic and anthropometric variables might facilitate assessment of completeness of a 24-hour urine collection also in hypertensive patients, as it was previously proposed in patients with CKD^20^. Our study therefore provides the first assessment of bias, precision, and accuracy of a number of available 24 hrUCr-estimating equations in hypertensive patients.

For several reasons, a perfect agreement between estimated and measured 24 hrUCr cannot be expected regardless of which formula is used. These include inherent imprecision of the equations, inherent variation of 24 hrUCr, and errors in urine collection for determination of actual 24 hrUCr. When serum creatinine level is at steady state, 24 hrUCr depends mainly on endogenous creatinine generation which is largely a function of muscle mass^21^ and varies by age, gender, body weight, race^13–16^, and other factors. Demographic and anthropometric variables are used in equations because they are readily available and correlate with muscle mass, the primary source of creatinine generation, but this correlation is imperfect. Muscle mass itself has also been included in some 24 hrUCr-estimating equations, either measured by dual-energy X-ray absorptiometry^21^ or taken into account more indirectly, e.g., as body cell mass measured by total body electrical impedance analysis^36^. However, in the study by Rule^21^, the model fit for 24 hrUCr estimated with age and gender was similar to that estimated with measured muscle mass. In addition, quantitative information on muscle mass is usually not routinely available in clinical practice. On the other hand, the importance of muscle mass as the major contributor to individual 24 hrUCr is highlighted by the fact that actually the major drawback of the best formulas in our study population was underestimation of 24 hrUCr in those with highest measured 24 hrUCr (perhaps reflecting the highest relative muscle mass), in whom the Gerber-Mann formula (which might have been developed in subjects with relatively high muscle mass) was in turn the best. Maybe different equations for those subjects with more vs. less muscular body physique could be offered to improve precision of individual 24 hrUCr estimates, and some recent data^36^ indicate that indirect estimation of muscle mass could offer some solution.

Despite approximability by the above demographic and anthropometric correlates, 24 hrUCr may show some variation in relation to other factors unaccounted for in the formulas, such as day-to-day variation in diet (protein intake), physical activity, and emotional stress^17,18^, and intraindividual diurnal, hour-to-hour, and day-to-day variation of urinary creatinine excretion rate was shown^37^. In addition, various disease states may have an effect on muscle mass. For example, patients with CKD have lower 24 hrUCr than those without CKD^38^, and 24 hrUCr may decline with progression of kidney disease due to increased extrarenal degradation of creatinine^39^.

Regarding generalizability/applicability of our findings, our study was performed in unselected hospitalized hypertensive patients who were subjected to clinical and diagnostic evaluation performed on a routine basis, with no special supervision over 24-hour urine and other clinical data collection. Therefore, our findings may be expected to be representative for the true accuracy of measuring and estimating 24 hrUCr in routine inpatient clinical practice. The patients in our study were admitted due to typical problems seen in a specialized inpatient hypertension service, mostly difficult-to-control hypertension or a suspicion of secondary hypertension. Thus, in our opinion the study population was generally representative for Caucasian patients with hypertension receiving specialist care, such as provided by hypertension specialists or specialist inpatient hypertension units, and our results may be generalized to such a population. The issue of the representativeness of our study population at a national level was discussed in more detail in our previous paper^12^.

### Study limitations

A limitation of estimating 24 hrUCr using body weight-based formulas is the expected difference in creatinine excretion between two subjects of the same weight, one of whom is more obese and the other more muscular. Thus, it can be expected that the estimated 24 hrUCr will be underestimated in a more muscular individual and overestimated in a more obese individual. We did not control for various factors potentially affecting creatinine generation/urinary excretion, such as diet and physical activity, but these factors may be expected to have the same effect on all formulas, and their variability is reduced in hospital settings. We did not include formulas without any anthropometric variables reflecting body size^40,41^, and formulas that include parameters that are often not routinely available, such as serum phosphorus^20^ and estimates of muscle mass^21,36^. Reference 24 hrUCr measurements were performed in a single 24-hour urine collection. Urine collection was not directly supervised and we cannot rule out collection errors. Para-aminobenzoic acid could not be used to evaluate completeness of 24-hour urine collection^2^ for logistic and financial reasons. However, in an attempt to reduce 24-hour urine collection errors, we only included patients with 24 hrUCr fulfilling the specific inclusion criteria. Finally, our results may not be representative for populations of other racial/ethnic characteristics.

### Summary and conclusions

In the present study, the performance of eight formulas for estimating 24 hrUCr based on demographic and anthropometric variables was compared in hospitalized hypertensive patients. We confirmed general utility of 24 hrUCr-estimating formulas in this population but also found significant differences between them, the best but not ideal being the CKD-EPI and Rule formulas. In contrast, the Gerber-Mann formula was the best in an analysis restricted to subjects with the highest measured 24 hrUCr in relation to body mass, a potential indicator of complete 24-hour urine collection but also a clear correlate of muscle mass. Strikingly different results obtained with the Mann-Gerber formula may be likely explained by restrictive inclusion criteria for the completeness of the reference 24-hour urine collection used for the development of this formula. Our findings indicate that if a 24 hrUCr-estimating formula is used in a population with different average 24 hrUCr compared to the population in which it was derived, the precision of estimates is significantly affected.

It can also be speculated that the issue of completeness of the reference 24-hour urine collections used for the development of various formulas was perhaps not adequately addressed in previous studies, and there is a potential to improve such formulas by addressing this factor. Finally, the observed agreement between estimated and measured 24 hrUCr indicates that in hypertensive patients, estimation of 24 hrUCr using formulas based on demographic and anthropometric variables may indeed facilitate assessment of completeness of 24-hour urine collections performed for any clinical reasons.

## Methods

The study population included 293 patients who were admitted to our specialist tertiary care hypertension unit and had a 24-hour urine collection performed. We included all patients in whom creatinine was measured in 24-hour urine collection and necessary demographic and anthropometric data (age, weight, and height) were available in the medical records. More information on the study protocol and conduct is available in a previously published paper^11^. In brief, all patients underwent clinical evaluation and diagnostic tests deemed necessary for the work-up of hypertension. The routine laboratory diagnostic protocol employed in our unit included a 24-hour urinary collection performed for various diagnostic reasons. Patients were asked for a 24-hour urine, instructed how to collect it and provided appropriate containers to collect urine themselves but the urine collection was not supervised by the hospital staff in regard to its completeness. The volume of collected 24-hour urine was measured by the laboratory staff, and creatinine level in samples taken from 24-hour urine collections was determined using standard laboratory methods in the central laboratory at our hospital (Cobas Integra analyzers, Roche). Results were expressed in mg/dL and multiplied by urine volume to yield measured 24 hrUCr in g/day. For estimating 24 hrUCr, eight formulas based on demographic and anthropometric variables (CKD-EPI, Cockcroft-Gault, Walser, Goldwasser, Rule, Gerber-Mann, Kawasaki, and Tanaka) were used, with respective equations shown in Table 3. Body surface area for the Rule formula was calculated using the Mosteller formula^42^.

**Table 3:** Formulas for estimating 24-hour urinary creatinine excretion.

Formula	Equation for 24 hrUCr (mg/24 hours)
CKD-EPI^20^	879.89 + 12.51 × weight (kg) − 6.19 × age + (34.51 if black) − (379.42 if female)
Cockcroft-Gault^13^	[28 − (0.2 × age)] × weight (kg) × 0.85 if female
Walser^14^	Men: (28.2–0.172 × age) × weight (kg) Women: (21.9–0.115 × age) × weight (kg)
Goldwasser^16^	[23.6 − (age/8.3) (+1.9 if black)] × weight (kg).
Rule^21^	{exp[7.26–0.26 (if female) − (0.011 × (age − 55) if age >55 years)]} × BSA/1.73 (m^2^)
Gerber and Mann* ^19^	699–421.9 if female + (16.83 × weight) (kg) − 25.82 (if white) − 2.67 × age
Kawasaki^22^	Men: −4.72 × age + 8.58 × weight (kg) + 5.09 × height (cm) − 74.5 Women: −12.63 × age + 15.12 × weight (kg) + 7.39 × height (cm) − 79.9
Tanaka^22^	−2.04 × age + 14.89 × weight (kg) + 16.14 × height (cm) − 2244.45

24 hrUCr, 24-hour urinary creatinine excretion; BSA, body surface area; CKD-EPI, Chronic Kidney Disease Epidemiology Collaboration.

*The original equation [699–421.9 if female + (7.64 × weight) (pounds) −25.82 (if white) − 2.67 × age (years)] transformed from weight in pounds to weight in kilograms.

The local Ethics Committee at our institution formally confirmed a non-interventional nature of our study and no requirement for patient informed consent due to the fact that all procedures in patients were undertaken as routine investigations indicated clinically, and samples were anonymized. The research was conducted in accordance to all the relevant national regulations, institutional policies and the tenets of the Helsinki Declaration.

To reduce bias related to potential 24-hour urine under- or overcollection, the main analysis only included patients in whom the measured 24 hrUCr in relation to body weight (expressed in mg/kd/d) was within the expected/reference range, serving as a measure of completeness of a given 24-hour urine collection. However, varying reference ranges for measured 24 hrUCr were reported in the literature, and to include the largest number of patients from our study population, for the main analysis we used the most liberal inclusion criteria we could identify in the literature, based on the reference ranges reported by the Mayo Clinic (men: 13–29 mg/kg/d, women: 9–26 mg/kg/d)^28^. However, other more restrictive reference ranges/inclusion criteria for measured 24 hrUCr were also reported in the literature or used in some studies in which the evaluated formulas were developed^19,29^. Of these, the reference ranges suggested by Imbembo and Walser (men: 15–25 mg/kg/d, women: 10–20 mg/kg/d)^29^ have apparently been more widely used in the previous studies^20,43^, while the inclusion criteria by Gerber and Mann (men: ≥20 mg/kg/d, women: ≥15 mg/kg/d)^19^ only set the lower threshold and thus select patients with the highest measured 24 hrUCr. Thus, as a sensitivity analysis, we also performed our analysis in the overall study population (all patients) and using more restrictive alternative inclusion criteria for the measured 24 hrUCr as suggested by Imbembo and Walser^29^ and by Gerber and Mann^19^.

The agreement between measured and estimated 24 hrUCr was assessed using the Pearson correlation coefficient and the Bland-Altman approach^23,24^. The latter involves calculation of the mean population difference between the two compared parameters, i.e., measured and estimated 24 hrUCr, which is referred to as the mean bias, and constructing a plot of individual differences between the two parameters against their individual mean in a given population. The mean bias value was calculated as the arithmetical mean of individual differences between measured and estimated 24 hrUCr, calculated for all study participants. The 95% limits of agreement for the Bland-Altman approach, which indicate the range where the error of individual estimation lies within in 95% of cases, were calculated as the mean bias value ± 1.96 standard deviation of the mean bias. We also visually assessed the slope of the Bland-Altman plot regression lines, showing whether a given formula remained similarly precise at the lower and upper end of 24 hrUCr range. To evaluate individual accuracy of the formulas, for each formula we also calculated the average of absolute differences between measured and estimated 24 hrUCr and the percentage of estimated 24 hrUCr values within 15%, 30%, or 50% of the measured 24 hrUCr (P15, P30, and P50, respectively).

### Statistical analysis

The R software was used for all calculations. The Friedman rank sum test, a non-parametric version of one-way analysis of variance with repeated measures, was used to compare differences between the mean bias values for the eight formulas. The bootstrap method^44^ (R software “boot” package, normal approximation) was used to calculate the 95% confidence intervals for the mean bias values. P < 0.05 was considered statistically significant.

## Supplementary information

Supplementary Table

Dataset

## Data Availability

All data generated or analysed during this study are included in this published article and its Supplementary Information files (Dataset.xlsx).
