# Supplementary material for: Performance of 24-hour urinary creatinine excretion-estimating equations in relation to measured 24-hour urinary creatinine excretion in hospitalized hypertensive patients
Source: Sci Rep. 2019 Mar 5;9:3593. doi: 10.1038/s41598-019-40416-w (PMC6401114; doi:10.1038/s41598-019-40416-w)
Supplement: Supplementary file 1 — Supplementary Table [file 41598_2019_40416_MOESM1_ESM.pdf]

Performance of 24-hour urinary creatinine excretion-estimating equations in relation to measured 24-hour urinary creatinine excretion in hospitalized hypertensive patients

Piotr Jędrusik, Bartosz Symonides, Zbigniew Gaciong

Supplementary Table. Comparative performance of 24-hour urinary creatinine excretion (24hrUCr)-estimating formulas in the overall study population (all patients) and using alternative inclusion criteria by Imbembo and Walser [29].

Formula	Bias (g/d)	95% CI	Absolute difference	R	95% LoA	P15	P30	P50
**Overall study population (all patients, n=293)**								
CKD-EPI	-0.091	-0.118 to -0.033	0.276	0.71	-0.790 to 0.608	45	73	86
Cockcroft-Gault	-0.045	-0.091 to 0.007	0.283	0.68	-0.801 to 0.710	46	71	88
Walser	-0.160	-0.200 to -0.105	0.299	0.69	-0.901 to 0.581	47	71	83
Goldwasser	-0.142	-0.181 to -0.081	0.327	0.61	-0.930 to 0.646	36	65	80
Rule	-0.052	-0.094 to -0.011	0.262	0.73	-0.721 to 0.616	49	78	88
Gerber-Mann	-0.401	-0.431 to -0.341	0.443	0.68	-1.146 to 0.344	28	48	68
Tanaka	-0.299	-0.343 to -0.251	0.386	0.66	-1.040 to 0.443	35	56	75
Kawasaki	-0.208	-0.259 to -0.168	0.314	0.72	-0.949 to 0.533	46	70	83
**Alternative inclusion criteria by Imbembo and Walser (n=206)**								
CKD-EPI	0.018	-0.014 to 0.047	0.164	0.89	-0.387 to 0.423	63	94	100
Cockcroft-Gault	0.063	0.022 to 0.092	0.197	0.85	-0.422 to 0.547	58	85	100
Walser	-0.049	-0.088 to -0.021	0.182	0.87	-0.496 to 0.398	63	90	100
Goldwasser	-0.043	-0.086 to -0.010	0.225	0.79	-0.583 to 0.497	46	81	95
Rule	0.024	-0.007 to 0.054	0.171	0.89	-0.397 to 0.446	64	96	99

Gerber-Mann	-0.286	-0.322 to -0.260	0.302	0.88	-0.703 to 0.131	34	60	86
Tanaka	-0.217	-0.252 to -0.182	0.269	0.85	-0.687 to 0.252	44	67	88
Kawasaki	-0.103	-0.141 to -0.079	0.200	0.89	-0.560 to 0.353	58	87	98

24hrUCr, 24-hour urinary creatinine excretion; CKD-EPI, Chronic Kidney Disease Epidemiology Collaboration.

Bias – measured minus estimated 24hrUCr; 95% CI – 95% confidence interval by the Friedman rank sum test; Absolute difference – average of absolute differences between measured and estimated 24hrUCr (g/d); R – Pearson correlation coefficient for estimated vs. measured 24hrUCr; 95% LoA – 95% limits of agreement by the Bland-Altman method (g/d); P15, P30, P50 – percentage of estimated 24hrUCr values within 15%, 30%, 50% of the actual measured 24hrUCr.
